# Risk factors for cardiovascular disease ten years after preeclampsia

**DOI:** 10.1590/S1516-31802010000100003

**Published:** 2010-01-07

**Authors:** Ivete Cristina Teixeira Canti, Márcia Komlós, Sérgio Hofmeister Martins-Costa, José Geraldo Lopes Ramos, Edison Capp, Helena von Eye Corleta

**Affiliations:** I MD. Postgraduate student on Medical Sciences program, Universidade Federal do Rio Grande do Sul (UFRGS), Porto Alegre, Rio Grande do Sul, Brazil.; II MD. Pediatric radiologist and Neuroradiology fellow, Cincinnati Children's Hospital Medical Center, Cincinnati, United States.; III MD. Postgraduate student on Medical Sciences program and assistant professor, Department of Gynecology and Obstetrics, School of Medicine, Hospital de Clínicas de Porto Alegre (HCPA), Universidade Federal do Rio Grande do Sul (UFRGS), Porto Alegre, Rio Grande do Sul, Brazil.; IV MD. Associate professor of postgraduate Medical Sciences program, Department of Gynecology and Obstetrics, School of Medicine, Hospital de Clínicas de Porto Alegre (HCPA), Universidade Federal do Rio Grande do Sul (UFRGS), Porto Alegre, Rio Grande do Sul, Brazil.; V MD. Associate professor of postgraduate Medical Sciences program, Department of Gynecology and Obstetrics, School of Medicine, and researcher, Gynecology and Molecular Obstetrics Laboratory, Hospital de Clínicas de Porto Alegre (HCPA), Universidade Federal do Rio Grande do Sul (UFRGS), Porto Alegre, Rio Grande do Sul, Brazil.

**Keywords:** Pre-eclampsia, Eclampsia, Cardiovascular diseases, Hypertension, Proteinuria, Pré-eclâmpsia, Eclampsia, Doenças cardiovasculares, Hipertensão, Proteinúria

## Abstract

**CONTEXT AND OBJECTIVE:**

Preeclampsia is a gestational disease that occurs mainly among nulliparous women after the 20^th^ week of gestation, and frequently close to delivery. The effects of preeclampsia on women's blood pressure over the long term are still controversial. Patients with recurrent preeclampsia or preeclampsia in the early stages of pregnancy appear to present higher risk of hypertension. The aim of this study was to determine the risk factors for cardiovascular disease among women with preeclampsia 10 years earlier.

**DESIGN AND SETTING:**

Cross-sectional study at Hospital de Clínicas de Porto Alegre (HCPA).

**METHODS:**

Forty women with preeclampsia and 14 normotensive pregnant women followed up 10 or more years earlier at HCPA underwent clinical and laboratory examinations. Spearman's correlation coefficient was used to correlate body mass index (BMI) and systolic and diastolic pressures. The risk of developing hypertension was measured using the chi-square test. P < 0.05 was considered significant.

**RESULTS:**

The patients with preeclampsia 10 or more years earlier had significantly higher diastolic blood pressure (P = 0.047), BMI (P = 0.019) and abdominal circumference (P = 0.026). They presented positive correlations between BMI and diastolic blood pressure (0.341; P = 0.031) and between BMI and systolic blood pressure (0.407; P = 0.009).

**CONCLUSION:**

The patients with preeclampsia 10 or more years earlier had significantly higher diastolic blood pressure, BMI and abdominal circumference than did the control group. This emphasizes the importance of long-term follow-up assessment for cardiovascular risk factors among patients with preeclampsia.

## RESUMO

**CONTEXTO E OBJETIVO:**

Pré-eclâmpsia é uma doença gestacional que ocorre principalmente em mulheres nulíparas, após a vigésima semana de gestação e frequentemente próximo do parto. Os efeitos da pré-eclâmpsia sobre a pressão sanguínea das mulheres a longo prazo ainda são controversos. Pacientes com pré-eclâmpsia recorrente ou pré-eclâmpsia na fase inicial da gestação parecem apresentar maior risco para hipertensão. O objetivo foi determinar os fatores de risco para doença cardiovascular em mulheres com pré-eclâmpsia há 10 anos.

**TIPO DE ESTUDO E LOCAL:**

Estudo transversal, realizado no Hospital de Clínicas de Porto Alegre (HCPA).

**MÉTODO:**

Quarenta mulheres com pré-eclâmpsia e 14 mulheres grávidas com pressão arterial normal acompanhadas há 10 anos ou mais no HCPA foram submetidas a exames clínicos e laboratoriais. Para se encontrar a correlação entre índice de massa corporal (IMC) e as pressões sistólica e diastólica, foi calculado o coeficiente de correlação de Spearman. O risco de desenvolvimento de hipertensão foi calculado através do teste de qui-quadrado. P < 0,05 foi considerado significante.

**RESULTADOS:**

Pacientes com pré-eclâmpsia há 10 anos ou mais tiveram pressão arterial diastólica (PAD) (P = 0,047), IMC (P = 0,019) e circunferência abdominal (P = 0,026) mais elevadas e apresentaram correlações positivas entre IMC e PAD (0,341, P = 0,031) e sistólica (0,407, P = 0,009).

**CONCLUSÃO:**

As pacientes com pré-eclâmpsia há 10 anos ou mais apresentaram pressão arterial diastólica, IMC e circunferência abdominal mais elevadas que o grupo controle. Isso enfatiza a importância de acompanhamento por longo tempo para avaliar fatores de risco cardiovascular em pacientes com pré-eclâmpsia prévia.

## INTRODUCTION

Preeclampsia is a gestational disease that occurs mainly among nulliparous women, particularly after the 20^th^ week of gestation, and most frequently close to the time of birth. It is diagnosed when the patient presents blood pressure of 140/90 mmHg or more, in two separate measurements at least four to six hours apart, and proteinuria (> 0.3 g/24 h).^1,2^ The incidence of preeclampsia is approximately 3-5%,^1,3,4^ although in some populations it may reach up to 10%.^5^ Its prevalence is also variable: 10 to 20%.^6^

The effects of preeclampsia on maternal blood pressure are still controversial^7^ Women with preeclampsia appear to have higher risk of developing hypertension in subsequent pregnancies. This risk is higher among nulliparous women with preeclampsia, in whom it starts earlier in the pregnancy (risk of up to 40%).^8,9^ The incidence of elevated systemic arterial pressure (SAP, i.e. diastolic arterial pressure ≥ 100 mmHg) was found to be 21.8% among women over 33 years of age who had had hypertension in a previous pregnancy, and only 10% (expected for the population) among women who had remained normotensive during pregnancy.^10^ Women who had had serious preeclampsia and those who had had eclampsia during their first pregnancy had significantly higher incidence of elevated SAP than shown by the general population, particularly when the period studied was longer than 10 years. Patients with recurrent preeclampsia and those in whom preeclampsia started during the early stages of pregnancy presented higher risk of future hypertension.^9^

Primiparae with preeclampsia who were evaluated 17 years after the pregnancy presented a positive association with insulin resistance and elevated blood pressure levels.^11^ Mild hyperinsulinemia, which may be a cause or consequence of preeclampsia, possibly contributes towards increased risk of cardiovascular disease (CVD) in the future, with higher likelihood of developing diseases such as diabetes mellitus (DM), high systemic arterial pressure (SAP) and ischemic coronary disease.^11,12^

Although the role of preeclampsia as another non-classical risk factors for CVD among women has become increasingly recognized,^13^ the traditional risk factors (positive family history of cardiovascular disease, low levels of physical activity, high BMI and central obesity) interact to compound the cardiovascular risk. The Framingham study model used age, smoking, systolic blood pressure and total and high-density lipoprotein cholesterol (HDL-c) to estimate the 10-year risk of CVD in women.^12^ Cardiovascular disease is one of the leading causes of death worldwide, and 80% of the cases occur in low-income and middle-income countries.^14^ The use of prediction rules or risk scores to identify women at higher risk is an established low-cost prevention strategy for prevention of cardiovascular disease.

## OBJECTIVE

The purpose of this study was to determine the risk factors for CVD among women with preeclampsia and/or eclampsia during pregnancies that occurred 10 or more years earlier.

## MATERIAL AND METHODS

### Study design

This was a cross-sectional study.

### Population and sample

Patients who delivered at the Gynecology and Obstetrics service of the Hospital de Clínicas de Porto Alegre (HCPA) 10 or more years before the time of the present study were selected. Patients who previously presented preeclampsia and normal pregnant women (control group) were selected according to their medical records. The study excluded patients who, at delivery (10 or more years earlier), had presented signs or a diagnosis of active or previous cardiovascular disease. The control group was composed of normotensive pregnant women who delivered on the same day as the women who comprised the study sample.

Invitation letters were initially sent to the selected patients. Those who came to the hospital were instructed about the study objectives, and they signed an informed consent statement. Informed consent was obtained from the patients who were interested in participating in the study. The following clinical data were obtained: age, race, parity, smoking habits, current diseases, physical activity, previous gynecological and obstetric history of preeclampsia, hypertension or diabetes mellitus, other previous obstetric diseases and any family history of risk factors for CVD. A physical examination was performed, with measurements of arterial pressure (two readings), weight, height, abdominal circumference and hip circumference. All of the patients underwent laboratory tests after 12 hours of fasting, which included: total cholesterol, triglycerides, high-density lipoprotein (HDL) cholesterol and low-density lipoprotein (LDL) cholesterol. Glucose levels were measured after 12 hours of fasting and two hours after consuming 75 mg of glucose. The data collection and physical examination were performed by two trained physicians. After obtaining the laboratory results, patients with abnormal findings were referred for any medical management needed.

This study was submitted to and approved by the Research Ethics Committee of the Research and Postgraduation Group of HCPA (GPPG 01-106).

### Statistical analysis

Statistical analyses were performed using the Statistical Package for the Social Sciences (SPSS) 13.0 statistical software. Clinical and metabolic variables were shown as mean ± standard deviation. The variables studied had normal distribution. Student's t test was used for comparisons between the control and preeclampsia groups and between smokers and nonsmokers. Spearman's correlation coefficient was used to correlate body mass index (BMI) and systolic and diastolic pressures. The risk of developing hypertension was measured by means of the chi-square test. The calculation of the risk of developing hypertension used the chi-square test. Statistical differences with a value of P < 0.05 were considered to be significant.

## RESULTS

In total, 54 patients who delivered at HCPA 10 or more years earlier were reexamined. The mean time elapsed since delivery was 14.6 ± 3.1 years in the control group and 15.9 ± 3.6 years in the preeclampsia group. The patients’ mean ages were similar at the time of drawing up this study: 37.2 ± 3.8 years in the control group and 39.2 ± 7.7 years in the preeclampsia group (P = 0.356). Other than antihypertensive drugs, just one patient in the control group was using thyroid hormone (levothyroxine).

The patients’ clinical and metabolic characteristics are shown in Table 1 (mean ± standard deviation and frequencies). The women in the preeclampsia group had significantly higher BMI, waist circumference and diastolic blood pressure than presented by the control group. All the other clinical and metabolic parameters were similar in the two groups. Table 2 shows the correlation between BMI and systolic and diastolic pressures (Spearman's correlation coefficient). The patients in the preeclampsia group presented a positive and statistically significant correlation between BMI and diastolic blood pressure (0.341; P = 0.031) and between BMI and systolic blood pressure (0.407; P = 0.009). The relative risk of developing arterial hypertension among the patients who had had preeclampsia was 2.43 (0.641-9.27). In the preeclampsia group, 15.0 % of the patients presented hypertension 10 years later, while in the control group, only 7.1 % of the patients did so.

**Table 1.** Clinical and metabolic characteristics

	Control		Preeclampsia		P	
	n = 14	Frequency of abnormal values	n = 40	Frequency of abnormal values
Number of pregnancies	3.29 ± 1.44		2.98 ± 1.46		0.495
Number of deliveries	3.00 ± 1.18		2.60 ± 1.22		0.289
Fasting glycemia (mg/dl)	93.27 ± 6.52	0%	91.15 ± 10.83	0%	0.430
OGTT 75 g (mg/dl)	112.09 ± 22.87	14.3%	115.23 ± 31.68	15%	0.718
Triglycerides (mg/dl)	105.18 ± 36.14	0	111.00 ± 82.19	7.5%	0.739
Total cholesterol (mg/dl)	200.54 ± 32.32	35.7%	196.00 ± 34.24	32.5%	0.691
HDL (mg/dl)	52.72 ± 10.24	0%	53.78 ± 12.03	0%	0.776
LDL (mg/dl)	126.81 ± 10.24	14.3%	120.66 ± 33.15	25%	0.557
BMI (kg/m^2^)	26.12 ± 4.53	57.1%	29.96 ± 6.13	75%	**0.019** *
Waist/hip ratio	0.827 ± 0.060	42.9%	0.856 ± 0.077	50%	0.157
Waist (cm)	84.92 ± 7.86	42.9%	93.15 ± 12.31	60%	**0.026** *
Systolic BP (mmHg)	112.30 ± 15.89	7.1%	121.00 ± 17.65	7.5%	0.110
Diastolic BP (mmHg)	71.53 ± 16.25	7.1%	82.00 ± 11.86	15%	**0.047** *

OGTT = oral glucose tolerance test; HDL = high-density lipoprotein; LDL = low-density lipoprotein; BMI = body mass index; BP = blood pressure.

Normal values: fasting glycemia, < 110 mg/dl; OGTT 75 g, < 140 mg/dl; triglycerides, < 150 mg/dl; total cholesterol, < 200 mg/dl; HDL, < 40 mg/dl is low and > 60 mg/dl is high; LDL, < 160 mg/dl; waist/hip ratio, 0.85; waist circumference, > 88 cm.

* Sample powers were estimated for the following variables: BMI (96.8), waist (98.6) and diastolic blood pressure (95.8).

**Table 2.** Correlation between body mass index (BMI) and systolic and diastolic blood pressures (Spearman's correlation coefficient)

BMI vs.	R	P
**Controls (n = 14)**		
Systolic BP	0.030	0.923
Diastolic BP	0.020	0.948
**Preeclampsia (n = 40)**		
Systolic BP	0.341	**0.031**
Diastolic BP	0.407	**0.009**

BP = blood pressure; R = Spearman's correlation coefficient.

The smoking habits were similar between the groups (P = 0.793). Analysis on the metabolic and clinical variables of smokers versus non-smokers showed that differed only in relation to HDL (43.9 ± 9.8 and 56.7 ± 10.3, respectively; P < 0.001). The abdominal circumference measurement did not show any correlation with the metabolic parameters.

## DISCUSSION

The consequences of preeclampsia/eclampsia for both the mother and the fetus are relatively well known. However, the long term consequences to the woman's health are not well understood. Although some studies have not demonstrated a higher risk of elevated SAP among patients with preeclampsia/eclampsia,^10,15^ other authors, while recognizing preeclampsia to be a manifestation of insulin resistance, have assumed that it involves a higher cardiovascular risk.^16,17^ Irgens et al. demonstrated that mortality caused by cardiovascular disease over the long term is 1.2 times greater among patients with preeclampsia than among those without it, and found an accentuated difference (8.2 times greater) among cases of acute preeclampsia (with preterm birth).^18^ In another retrospective study, women who had had preeclampsia 15 to 19 years earlier, presented twice as much chance of hospital admission and death due to ischemic heart disease as did pregnant women without preeclampsia.^19^ More recently, a Canadian retrospective study^21^ on 1.03 million women identified a risk of CVD (coronary revascularization, stroke and peripheral arterial disease) that was twice as great among pregnant women with some type of placental syndrome (hypertensive gestational disease, placental abruption or preeclampsia) than among pregnant women without placental syndromes. Other authors have correlated preeclampsia with more frequent cardiovascular disease.^11,16,18,20,22^

Significantly higher diastolic pressure presented in the group of women with preeclampsia/eclampsia, compared with the control group, was also described by Wilson et al.^23^ and Forest et al.^24^ The BMI and abdominal circumference measurements were significantly higher among women in our study who had had preeclampsia/eclampsia, and this was thought to indicate centripetal distribution of fat, since the waist/hip ratio did not differ. Abdominal fat distribution is also an early risk factor for cardiovascular disease, hypertension^25^ and insulin resistance^26^ in this group of patients.

There was a positive correlation between BMI and systolic blood pressure (0.341; P = 0.031) and between BMI and diastolic blood pressure (0.407; P = 0.009) in the group of patients with preeclampsia. Such findings were also described by Forest et al.^24^

There was no difference in the prevalence of diabetes mellitus and elevated SAP between the patients with preeclampsia and the controls, and this might be explained by the age difference between the study groups (women with preeclampsia: 39.2 ± 7.7 years old; and control group: 37.2 ± 3.8 years old).

Finally, despite our efforts, a great number of the patients had moved away and could not be contacted after 10 years. Considering the variable of diastolic blood pressure and assuming a type 1 error of P = 0.047, this sample had a statistical power (type 2 error) of 95.8 to detect the difference found. For BMI, assuming a type 1 error of P = 0.019, the statistical power (type 2 error) was 96.8; and, for waist circumference, assuming a type 1 error of P = 0.026, the statistical power (type 2 error) was 98.6. For the other variables, our sample did not have enough statistical power to detect differences.

## CONCLUSIONS

Patients who had had preeclampsia 10 or more years earlier presented significantly higher diastolic blood pressure, BMI and abdominal circumference measurements than did those in the control group. These simple and low-cost anthropometric measurements could identify which women with preeclampsia might benefit from greater rigorousness of clinical tracking and perhaps an early intervention in relation to cardiovascular diseases. Therefore, we believe that abdominal circumference and BMI measurements should form part of the routine clinical evaluation for patients with a prior history of preeclampsia or eclampsia, in order to decide on the most appropriate preventive measures and management for potential cardiovascular complications.
